# In Vitro Human Liver Model for Toxicity Assessment with Clinical and Preclinical Instrumentation

**DOI:** 10.3390/pharmaceutics16050607

**Published:** 2024-04-29

**Authors:** Eneko Madorran, Lidija Kocbek Šaherl, Mateja Rakuša, Miha Munda

**Affiliations:** Faculty of Medicine, Institute of Anatomy, Histology and Embryology, University of Maribor, Taborska Ulica 8, 2000 Maribor, Slovenia; lidija.kocbek-saherl@um.si (L.K.Š.); mateja.rakusa@um.si (M.R.); miha.munda@um.si (M.M.)

**Keywords:** liver in vitro model, in vitro toxicity, translational research, novel biomarkers

## Abstract

The existing in vitro toxicological models lack translational potential, which makes difficult the application of gathered information to clinical usage. To tackle this issue, we built a model with four different types of primary liver cells: hepatic sinusoidal endothelial cells, hepatic stellate cells, Kupffer cells and hepatocytes. We cultured them in different combinations of composition and volumes of cell medium, hepatocyte proportions of total cells and additions of extracellular matrixes. We added rifampicin (RIF), ibuprofen (IBU) and 5-fluorouracil (5-FU) to this model and observed the microanatomy and physiology changes for a week with preclinical and clinical instruments. Among the different model configurations, we selected the feature combination of the in vitro model that had similar biomarker values to those measured in clinical diagnostics. When we exposed the selected model configuration to RIF, IBU and 5-FU, we observed similar glucose, triglyceride and albumin dynamics as in vivo (from clinical data). Therefore, we have built an in vitro liver model that resembles the liver microenvironment, and we have analysed it with clinical instrumentation to facilitate data translation. Furthermore, during these observations, we found that Kupffer and LSEC cells are suitable candidates for the search for clinical diagnostic markers of liver function.

## 1. Introduction

Assuming that an organ model recreates the in vivo microenvironment accurately, any toxicity assessment in such model should be accurate. Under these assumptions, numerous new organ models have been developed to test the safety of drugs [1,2,3]. Two-dimensional hepatocyte models have the advantage that there are relevant and primary human hepatocytes that are recognised to be the ‘gold standard’ for in vitro hepatotoxic assays [1]. Yet, they have a short lifespan, and they lack other cell types present in the liver that are essential for the liver’s normal functioning [4]. The liver organ-on-a-chip (Ooc) models showed promising results [3], but they are designed with a limited number of cells and low cell densities in comparison to the in vivo environment [4]. The spheroid liver models have a greater cell number than the Ooc models and resemble most closely the in vivo liver microenvironment [2]. But the cells in the middle of the spheroid lack nourishment, and the cell density is lower than the in vivo microenvironment [4]. 

Thus, there are many different liver in vitro models, but none of the existing models had the characteristics that we considered critical for toxicity testing. Moreover, the preclinical and clinical endpoints are essentially too far apart to facilitate the translation of preclinical studies to clinical usage and may lead to paradoxical results in the toxicity assessment of substances [4,5,6,7,8]. 

In this sense, we designed an in vitro liver model based on the characteristics previously proposed by Madorran et al. (combinations of the composition and the volume of cell medium, the hepatocyte proportion of total cells, and the addition of extracellular matrixes) [4]. In addition, we used preclinical and clinical instruments to measure the physiology and anatomy of the built model to favour the comparison between the values we measured in the model with the clinical data from the literature (clinical cases). Under these premises, we were able to build a cell-based model that has similar values to that observed in clinical cases, and therefore building a liver model that recreated the in vivo liver microenvironment. At the same time, we uncovered promising targets that may be used as liver functions’ early markers in clinical diagnostics.

## 2. Materials and Methods

### 2.1. Cell Culture Medium

We used two different cell culture media to culture the cells alternatively:

i. Williams E colourless medium (Thermo Fisher Scientific, Waltham, MA, USA) containing 10% foetal bovine serum (FBS) (Thermo Fisher Scientific, USA). L-glutamine (2 mM, Sigma, Saint Louis, MO, USA), penicillin (100 U mL^−1^, Sigma) and streptomycin (1 mgmL^−1^, Fluka, Buchs, Switzerland) were also added for optimal cell growth;
ii. A medium based on Williams E’s colourless medium, which we will refer to from now on as Hep medium (Supplementary Table S1). This medium was supplemented with additional amino acid, fatty acids, vitamins and insulin (detail description in Supplementary Table S1). We used Maxgel, a commercial extra cellular matrix (ECM) (Merck KGaA, Darmstadt, Germany).

### 2.2. Cells

The liver model was built by coculturing hepatocytes from Lonza (Switzerland) and nonparenchymal liver cells (NPCs) from (ZEN-BIO, Durham, NC, USA): liver sinusoidal endothelial cells (LSECs), human stellate cells (HSCs) and Kupffer cells. NPCs and hepatocytes were grown separately in a 25 cm^2^ flask (NUNC, Roskilde, Denmark) in a controlled environment at 37 °C and 5% CO_2_ and later seeded together in a 96-well microplate (NUNC, Denmark) to build the liver models. At this point, we cultured them for 5 days.

We seeded the cells in two different seeding arrangements. In the first configuration, we seeded 10,000 hepatocytes and 2500 NPCs in each well (80% hepatocytes of total cells). In the second seeding arrangement, we seeded 10,000 hepatocytes and 6600 NPCs (60% hepatocytes of total cells).

In the following experimental setup (when exposing the model to hepatotoxic drugs), more cells were seeded in each sample: 20,000 hepatocytes and 5000 NPCs.

### 2.3. Toxic Agents

Rifampicin (RIF), ibuprofen (IBU) and 5-fluorouracil (5-FU) (Merck KGaA, Darmstadt, Germany) were added to the two cell culture media at the following final concentrations: 50 µmol/L RIF, 1 mmol/L IBU or 500 µmol/L 5-FU.

### 2.4. Analytical Techniques 

Three different analytical techniques were used to evaluate the physiology and anatomy of the model: the Zeiss Axiovert 40CFL inverted microscope (Zeiss, Oberkochen, Germany), the Cobas C111 biochemical analyser (Roche, Basel, Switzerland) and the Imagestream MK2 (ISX) imaging flow cytometer (Luminex, Austin, TX, USA).

### 2.5. Statistical Analysis

All statistical analyses in this study were performed using the R program. We determined any statistical differences among the features’ influence in the viability or the biomarker values of the samples with the Wilcoxon signed-ranked test. We evaluated the differences in viability and biomarkers between the in vitro liver-model samples treated with hepatotoxic drugs with the ANOVA/Tukey HSD test.

## 3. Results

In the Section 3.1, we analysed the effect of the various feature combinations we selected in our liver model because there were no previous data regarding their effect. After the analysis, we selected the feature combination that best represented the liver microenvironment and microanatomy and presented the results of these observation in Section 3.2.

### 3.1. Analysis of the Liver Model with Different Feature Combinations

**Figure 1:**
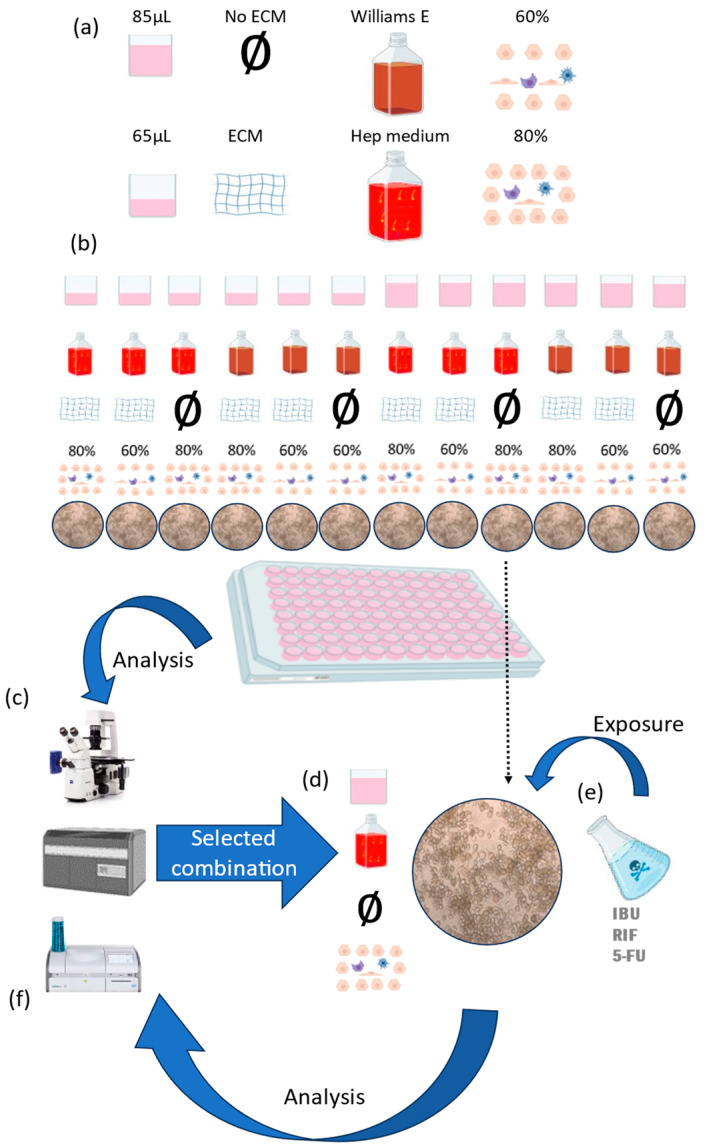
Schematic presentation of the experimental protocol. (**a**) Cells were cultured with different cell culture media formulations and volumes, ECMs and cell percentages of the different cell types. (**b**) Table summarizing the samples with their feature combinations (each combination was cultured in parallel). (**c**) All the combinations were analysed with the inverted microscope, the biochemical analyser and ISX. (**d**) The feature combination that most resembled the liver microenvironment was selected and (**e**) was exposed to IBU, RIF and 5-FU. (**f**) The same analysing methods were used to determine the toxic effect of the drugs in the liver model with the selected feature combination.

We observed the cell morphology, cell viability and clinical biochemical markers in the liver model we built with different features. We tested different feature combinations: we cultured the cells with different cell culture volumes (65 µL and 85 µL), different cell culture media compositions (Williams E medium and Hep medium), different hepatocyte densities (80% and 60%) and the addition of an ECM (some samples with and some without it) (Figure 1a).

#### 3.1.1. Cell Morphology

**Figure 2:**
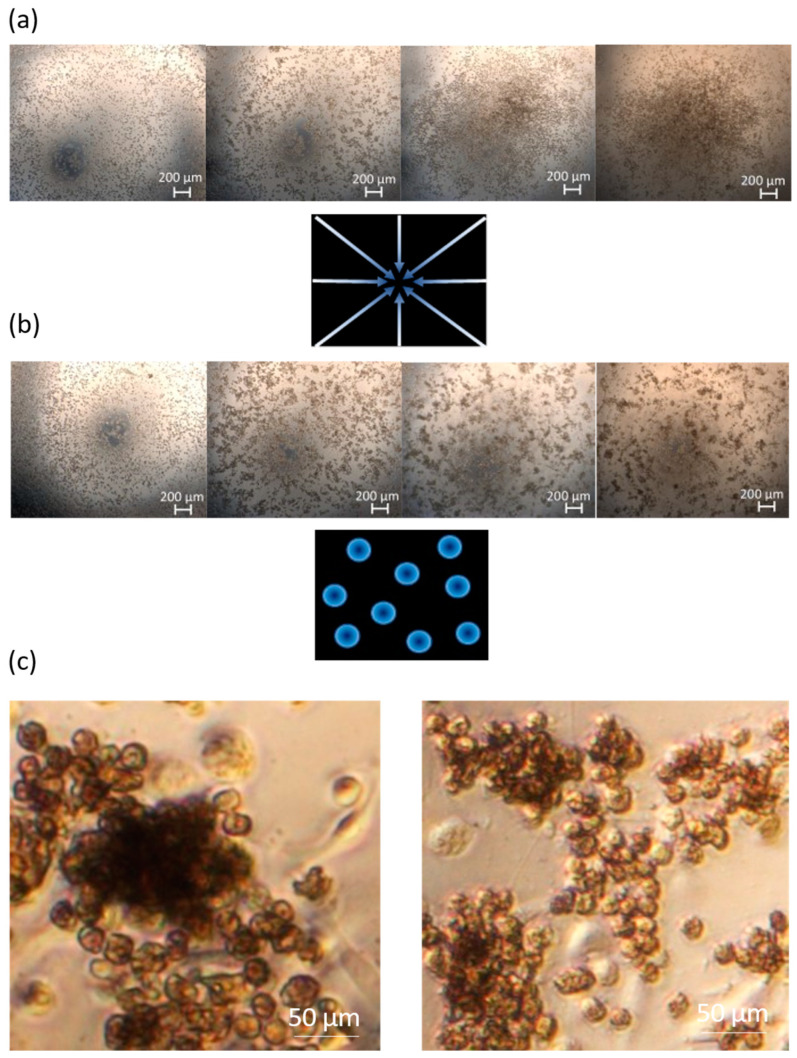
Observations of cell arrangement and morphology with the inverted microscope. (**a**) A sample cultured with 65 µL, in which the cells have migrated towards the centre (5× magnification). (**b**) A sample cultured with 85 µL, in which the cells have formed scattered colonies (5× magnification). In both cases, (**a**,**b**), we observed the transition of the cells from day 1 to day 5 in a 96-well microplate. (**c**) On the left, cells cultured with ECMs and on the right, cells cultured without ECMs (20× magnification).

We observed two important cell arrangements under the inverted microscope. The cells cultured with 65 µL of the medium clustered in the centre of the well (Figure 2a). The cells cultured with 85 µL of medium formed colonies on the entire surface of the well (Figure 2b). We did not observe any morphological differences between the samples cultured with or without ECMs (Figure 2c). It is noteworthy that the cells form similar structures to those observed in liver spheroid models (Figure 2c).

#### 3.1.2. Cell-Viability Assessment

**Figure 3:**
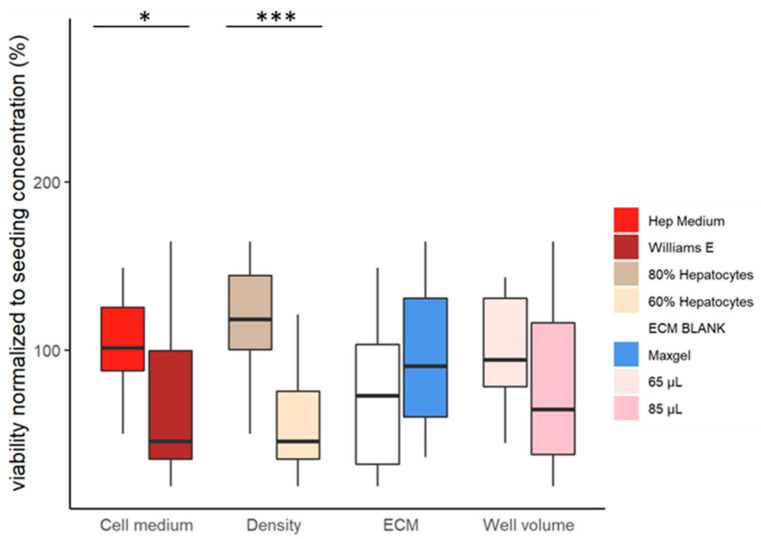
Viability of cells concerning the different combinations of features (*p*-value > 0.05, <0.05 * and <0.001 ***).

The models with the highest hepatocyte density (80%) had the highest cell viability (Figure 3). On the other hand, the models with the lowest hepatocyte density (60%) had the lowest cell viability (Figure 3).

The formulation of a cell medium also influences the viability of the models. Cells cultured with a Hep medium had significantly higher viabilities than cells cultured with William E (Figure 3).

#### 3.1.3. Clinical Biochemistry

The value of each liver marker was subtracted from the liver values measured in both cell culture media (without the cells). These values are summarized in Supplementary Table S2. We analysed alanine aminotransferase (ALT), aspartate aminotransferase (AST), gamma-glutamyl transferase (GGT), alkaline phosphatase (ALP), glucose, triglycerides and albumin in the pooled samples using a Cobas C111 (Roche, Switzerland) [9].

**Figure 4:**
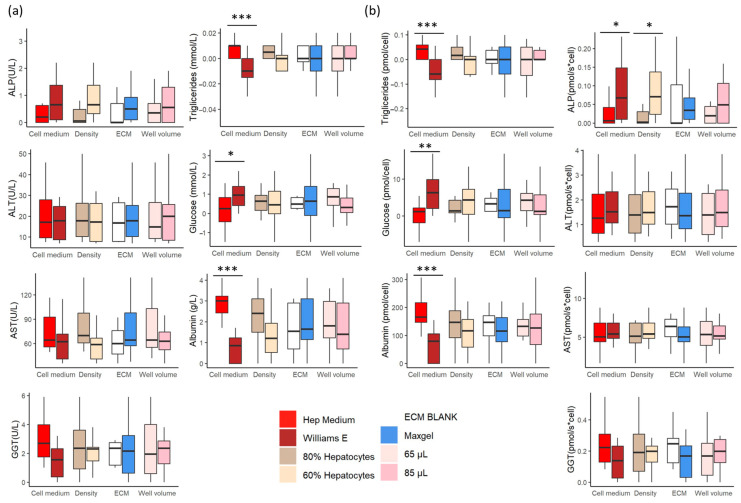
Values of the liver markers in the various liver models built with different feature combinations. (**a**) Liver-marker concentrations. (**b**) Liver-marker values normalised to cell number (molecule biomarker per cell). (*p*-value > 0.05, <0.05 *, <0.01 ** and <0.001 ***). We analysed alanine aminotransferase (ALT), aspartate aminotransferase (AST), gamma-glutamyl transferase (GGT), alkaline phosphatase (ALP), glucose, triglycerides and albumin.

We observed a significant difference in liver-marker values between the two cell culture media (Figure 4a, red and brown boxplots). Significantly higher albumin levels were measured in the samples cultured with the Hep medium. In addition, the cells cultured in this medium had a higher net triglyceride release. In contrast, cells cultured with the Williams E medium had a higher net uptake of triglycerides. The glucose dynamics were significantly different (*p* = 0.00024) and had an opposite effect to the triglyceride dynamics.

We did not observe any significant differences between the rest of the samples (Figure 4a). However, the normalisation of the values of each biomarker with the cell number of the corresponding sample revealed significant differences (Figure 4b). The normalisation showed a significantly lower ALP value in the samples cultured with the Hep medium (Figure 4b). It also showed a significantly lower ALP value in samples with higher hepatocyte densities.

### 3.2. Testing the Selected Model with RIF, IBU, 5-FU

Based on the previous observations, we selected the liver configuration that best represented the liver microenvironment (Figure 1d): 85 µL of Hep medium with 80% hepatocyte density and no added ECM. We perturbed the cells with RIF, IBU and 5-FU in the selected model and analysed them with the same tests as in Section 3.1.

#### 3.2.1. Morphology Assessment

**Figure 5:**
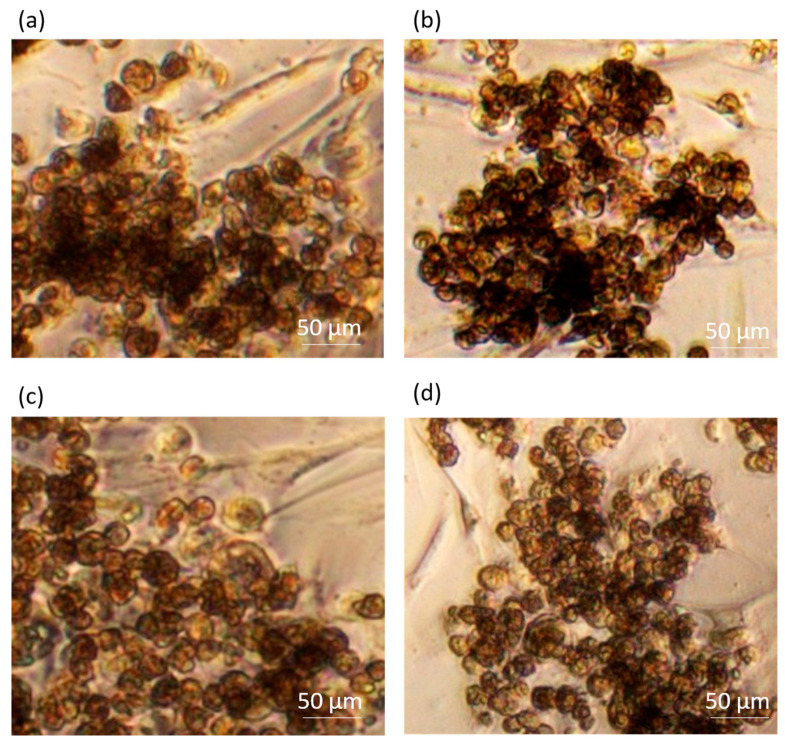
Evaluation of cell morphology with the inverted microscope (20× magnification) on each cell treatment. (**a**) Untreated, (**b**) RIF, (**c**) IBU and (**d**) 5-FU.

There were no visible morphologic differences between the groups in the first 5 days (Figure 5). Longer incubation periods should be performed to observe possible morphological changes. We did not perform longer incubations, since the focus of our study was to culture a cell-based liver model for 5 days.

#### 3.2.2. Viability Assessment

**Figure 6:**
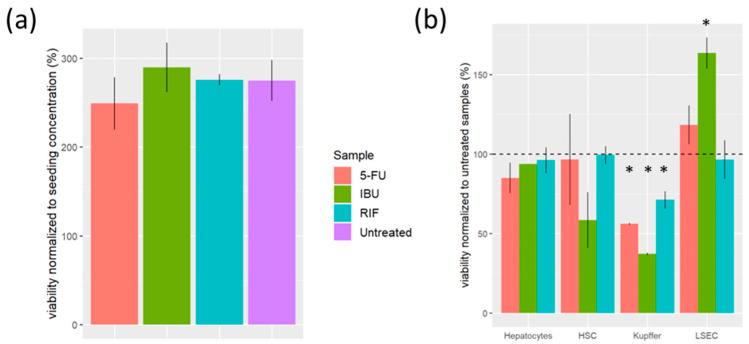
Viability of samples treated with RIF, IBU and 5-FU. (**a**) Viability of samples relative to the initial seeding number. (**b**) Viability of each cell type normalised to the untreated sample (*p*-value > 0.05, <0.05 *). Rifampicin (RIF), ibuprofen (IBU), 5-fluorouracil (5-FU), liver sinusoidal endothelial cells (LSECs) and human stellate cells (HSCs).

We did not observe any significant differences in the viability of the samples treated with 5-FU in respect to the untreated samples (Figure 6a). However, when analysing the viability of each cell type of the sample, we observed that Kupffer cells had the lowest viability (Figure 6b). In contrast, exposure to 5-FU induced the LSEC to proliferate during the first week (Figure 6b). However, we observed a high cell-death ratio on day 5 (Table 1). So, the continuous exposure of 5-FU to LSEC cells may reduce their viability.

We observed an ambivalent effect on the viability of cells exposed to IBU (Figure 6b). On the one hand, we observed that the viability of Kupffer and HSC cells was lower when the samples were exposed to IBU compared to the untreated samples (Figure 6b). On the other hand, we observed a higher viability of LSEC cells exposed to IBU (Figure 6b).

We did not observe any significant differences in the viability of the samples exposed to RIF (Figure 6a). Yet, the viability of the Kupffer cells was lower than that of untreated cells (Figure 6b).

#### 3.2.3. Clinical Biochemistry

**Figure 7:**
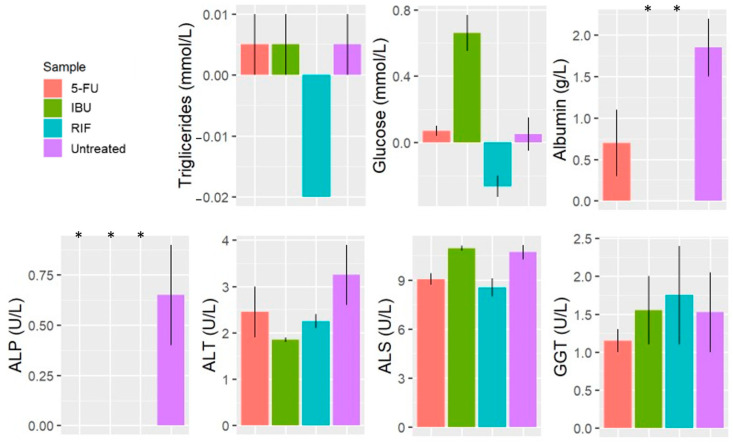
Clinical markers of samples treated with RIF, IBU and 5-FU in the selected liver model (Figure 1d). (*p*-value > 0.05, <0.05 *). Rifampicin (RIF), ibuprofen (IBU) and 5-fluorouracil (5-FU).

We observed a significant decrease in the ALP value for all treated samples compared to untreated samples (Figure 7). We also observed a lower albumin content in treated samples, but the decrease was significant only in samples treated with IBU and RIF.

We also observed that samples exposed to IBU had a significantly higher net release of glucose than the untreated sample (Figure 7). On the contrary, there was a net uptake of glucose and triglycerides in cells treated with RIF (Figure 7).

ALP, ALT, AST and GGT were similar in all the samples, and no significant effect was observed when treating the model with RIF, IBU nor 5-FU (Figure 7).

## 4. Discussion

Our goal was to build an in vitro liver model to resemble the liver microenvironment and allow for clinical-marker usage. To that end, we used primary liver cells, HSC, LSEC, Kupffer and hepatocytes, because they reportedly retain the characteristics of the original cells, unlike cancer cells or immortalized cell lines [10]. We also used different combinations of cell medium volume and formulation, ECM addition, and cell-type proportion to configure a liver model. In this sense, we evaluated the influence of each feature in the liver model.

### 4.1. The Influence of the Features in the Liver Model

We did not find any reference to the use of clinical instrumentation to analyse an in vitro liver model. Likewise, we did not find any in vitro liver model with these feature combinations. Therefore, we wanted to analyse the impact of these features on the microanatomy, physiology and viability of the model.

#### 4.1.1. The Influence of Cell Culture Media

We observed that cells cultured with a medium rich in supplements (Hep medium) increased the viability of the cells (Figure 3), which is concordant with previous studies [11,12]. The addition of insulin to the medium increased the glucose uptake by the cells (Figure 4), as described in the literature [13,14,15]. In contrast, the net release of triglycerides observed in cells cultured with the same media may be due to the higher amino acid content of the media [16]. The opposing dynamics of glucose and triglycerides (Figure 4a) are consistent with in vivo observations [16]. The addition of amino acids in the Hep medium increased the albumin synthesis significantly (Figure 4a), which concurs with the literature [17].

#### 4.1.2. The Influence of Cell Culture Volume

In the initial phase, the higher cell density and interaction in samples cultured with 65 µL of cell medium (due to the cell arrangement seen in Figure 2a) may have increased cell proliferation [18]. However, cell necrosis due to nutrient deficiency may have occurred later [10,19]. Taking these facts into account, as well as the property of a lobular arrangement of the liver [20], the cells cultured with 85 µL more closely resemble the in vivo situation (Figure 2b), as they formed scattered colonies resembling lobule-like arrangement. There are many different types of stimuli that induce cell migration [21]. But, considering that the different migrations we observed were related to cell density, we associate the migration with chemotaxis [22]. This complex process needs to be studied in much more detail to better understand the observed migrations.

Furthermore, the clinical instrumentation is designed to measure the markers at certain concentration ranges. However, the concentration of the molecules of interest is different in in vitro models and in in vivo environments. Thus, lowering the volume to increase the cell-number-to-cell-volume ratio enabled the use of clinical instrumentation.

#### 4.1.3. The Influence of ECM

The addition of ECMs did not affect the morphology of cells in the model (Figure 2c). On the contrary, the addition of ECMs had a noticeable impact on the viability of the cells. ECMs induced the proliferation of hepatocytes (as previously observed by Wang Y. et al. [23]) but decreased the viability of HSCs. The designed model involved HSCs responsible for synthesising ECMs in the liver [24]. Thus, the ECM production of the existing HSCs was sufficient [24], and the additional ECM coating did not affect any of the biomarker values.

#### 4.1.4. The Influence of the Proportion of the Cell Types

Higher hepatocyte density (80%) induced cell proliferation (Figure 3), which was previously observed [18]. These samples (80% hepatocyte density) had less variable glucose contents (Figure 4a), as expected, since hepatocytes are the liver cells that regulate glucose levels [16]. The samples with higher hepatocyte densities also had higher albumin contents (Figure 4a), as albumin is only synthesised in hepatocytes [25].

### 4.2. Testing the Selected Model

After evaluating the influence of each feature in the model, we selected the feature combination that most faithfully recreated the liver microenvironment and exposed the liver model to 50 µmol/L RIF, 1 mmol/l IBU or 500 µmol/L 5-FU. The concentrations chosen are based on previous studies by other authors [26,27,28], analysing cell proliferation, IC50, EC50, gene expression, CYP activities and data from our own experiments. We compared the effects of these drugs in the microanatomy, physiology and viability of the model with the existing clinical data. Thus, we could observe if the drugs induced a similar toxicity response in our liver model as documented in clinical data from patients treated with the same drugs.

#### 4.2.1. Exposing the Liver Model to 5-FU

5-FU is a widely used chemotherapeutic drug and one of the most commonly utilized drugs for the treatment of various types of cancers because it inhibits thymidylate synthase [29]. The hepatotoxicity of this drug is well documented [27,29,30,31]; thus, it is interesting as a toxic agent for liver models. The samples exposed to 5-FU had the lowest viability of all the evaluated samples (Figure 6a) because only 5-FU was cytotoxic to hepatocytes (Figure 6b). Since hepatocytes account for the vast majority of the population, any change in their cell number induces a higher effect in the total cell number of the sample. Yet, the highest cytotoxic effect was evaluated in Kupffer cells. In contrast, 5-FU induced the proliferation of LSEC cells during the first week, for which there are no previous data in the literature. However, given the high rate of cell death observed on day 5 (Table 1), continuous exposure to 5-FU may also induce cell death in LSEC cells [32]. The inhibition of thymidylate synthase and the accumulation of toxic by-products from 5-FU catabolism (fluorocitrate, for instance) may induce this delayed toxic effect [29]. We also observed that adding 5-FU to the model decreased albumin synthesis. This is more likely due to the reduction in hepatocyte numbers [33].

#### 4.2.2. Exposing the Liver Model to IBU

IBU is a nonsteroidal anti-inflammatory drug and one of the most used drugs worldwide [34]. There are many animal studies that have observed the hepatotoxicity of this drug [35,36,37,38], and considering its exposure [34], it is an interesting candidate for the evaluation of our model. The addition of IBU had an ambivalent effect on the cell viability of the samples. On the one hand, IBU significantly decreased the viability of Kupffer and HSC cells (Figure 6b). On the other hand, samples with added IBU had twice as many LSEC cells as the untreated samples (Table 1). The proliferation of LSEC was also observed in a previous study [39], yet the mechanisms involved are unknown to date. As a result of both trends (cytotoxicity and proliferation), the samples exposed to IBU had a similar viability to the untreated samples, but the cell composition of the liver model changed (Figure 6b). Yet, the most significant biological response induced by IBU was related to glucose dynamics. The samples treated with IBU had a significantly higher net glucose release than the untreated samples. And, these higher values were not the result of a greater net triglyceride uptake. Thus, the influence of IBU on gluconeogenesis and glycogenolysis was also observed in vivo [35,40,41] and could be the cause of this observation. Notably, there was no albumin content in samples treated with IBU, which may be caused due to the higher net glucose release (and lower availability for albumin synthesis) [33].

#### 4.2.3. Exposing the Liver Model to RIF

RIF is a widely used antibiotic to treat tuberculosis and other bacterial infections. Treatment with RIF is effective, but is known to induce drug-metabolizing enzymes in the liver [42]. Thus, it is an interesting compound to evaluate the toxic assessment of our model. The addition of RIF significantly reduced the viability of Kupffer cells, like in the rest of the treated samples (Figure 6b). On the contrary, RIF addition had a very distinct effect on glucose and triglyceride dynamics. Both markers had a significantly higher net uptake by the cells compared to the untreated samples (Figure 7). This is in line with different studies reporting fatty acid accumulation upon RIF treatment [43]. Moreover, this observation could also indicate an increase in FA accumulation within cells [16]. This evaluation is consistent with previous studies, in which the upregulation of the free fatty acid transporter was observed in HepG2 cells treated with 10 µM of RIF [43]. The albumin synthesis was significantly reduced when exposing the cells to RIF, maybe due to the above-mentioned changes in the molecular pathways involved in glucose and triglyceride dynamics [17]. Although, further investigation is needed to corroborate the latter.

### 4.3. Evaluation of the Liver Model

The model has a sufficient cell density to be statistically relevant, unlike Ooc models [44], and it has a cell arrangement similar to that in spheroids, which reinforces the native physiology of the cells [2]. It is visible under the microscope, allowing for the observation of detailed cell morphologies. It can also be analysed with clinical instruments, which facilitates comparisons with clinical data. In addition, the model enables comprehensive cell analysis with preclinical instruments. Most importantly, our observations of the model suggest that it resembles the liver microenvironment. The liver regulates the dynamics of triglycerides and glucose, which are in an inverse relationship [16,41], and these conditions are met in the model we developed (Figure 4a). Furthermore, the addition of hepatotoxic drugs to the model resulted in similar physiological responses as in vivo. Albumin synthesis is a key element in any liver model because albumin production is one of the liver’s main functions, and changes in albumin synthesis are associated with various pathologies [17,45]. Therefore, in our model, we observed the influence on albumin synthesis of hepatotoxic drugs (Figure 7).

In the tested liver models, we did not observe any correlation between transaminase values and the viability of the cells. But, the influence of transaminase levels in the liver is not clear in clinical studies either. High liver transaminases are found in patients whose liver is proliferating (after liver recession) [46]. But, various studies have measured similar levels in patients with liver diseases [47,48,49,50,51]. In addition, clinical studies have observed patients with abnormal levels who did not have liver disease [50,51]. These observations have led other authors to search for alternative markers in recent years [52]. Our model may aid in the search for alternative markers, which has been a rising concern in recent years [53,54,55]. Moreover, this model may shed further light on some specific pathology-related features, since it allows for a more concrete analysis using preclinical evaluation methods besides the use of clinical instrumentation. To that end, our study may uncover an underlying trend that was unclear earlier on. We observed that all hepatotoxic drugs had a greater impact on the viability of LSEC and Kupffer cells than on HSC and hepatocytes. Previous studies have observed transcriptomic shifts between healthy and cirrhotic liver disease (CLD) scenarios [56,57] on LSECs. But, they did not focus on the viability of both cell types. This finding is supported by the function of both cells, since LSECs and Kupffer cells are the liver cells responsible for xenobiotic uptake [58]. Thus, LSECs and Kupffer cells are the liver cells first affected by exogenous agents [58]. Therefore, we can use this model (or similar) to monitor molecules related to LSEC and Kupffer injury.

### 4.4. Limitations of the Model

In this experimental setup, we observed the cells in the model with an inverted microscope, but we are working on the next experimental setup, where we will observe the model with a confocal microscope. The use of a confocal microscope for further studies should allow for us a more detailed study of the anatomy of the cells in this model.

With the current settings, the model is not suitable for pharmacokinetic studies, but key changes in its configuration may make it adequate for such studies. The introduction of a rocker or even perfusion is possible and would enable pharmacokinetic studies.

It should also be interesting to culture the model with alternative cell culture media, especially media that may induce pathophysiological changes in the model (fatty acid-rich medium to induce steatosis).

In this study, we focused on the microanatomy, physiology and viability of the cells in the liver model. However, questions related to the molecular biology of the model should also be analysed in depth. Examples include, but are not limited to, the urea regulation or CYP expression of hepatocytes, the expression of scavenger receptors in LSECs, HSC retinoate-storing capabilities, the state of Kupffer cells, etc. Future research should prioritize this approach, especially when looking for potential liver markers, as we will discuss below.

The use of clinical instrumentation to evaluate the model was troublesome, due to the scarcity of information on the use of clinical instruments in an in vitro liver model. In this sense, more replicates under similar conditions should be performed to increase confidence in the observed trends. Special focus should be drawn to molecules present in LSECs and Kupffer cells in the model when exposing it to toxic compounds. If these molecules were also present in the peripheral circulation, they may be used as markers of liver health.

## 5. Conclusions

The developed model allows for a comprehensive evaluation of the toxicity of substances, since it is possible to observe with preclinical and clinical instruments. Moreover, when exposing the model to hepatotoxic drugs, we observed similar values to the data available from clinical diagnostics. Thus, this should aid in better comparisons between the data from preclinical and clinical observations. At the same time, it contributes to a better understanding of the physiology and anatomy of the human body. In this sense, a possible underlying physiological trend can be observed in the evaluation of toxicity, since LSECs and Kupffer cells were the only cell types that showed a measurable biological response. Thus, both cell types are reasonable candidates as clinical diagnostic markers of liver function, and we recommend further studies to substantiate our findings.

## Figures and Tables

**Table 1:** Cell-death ratio and population share of each cell type 5 days after exposing the cell-based liver model to hepatotoxic drugs.

Group	Share of the Population (%)	Cell-Death Ratio (%)	Share of the Population (%)	Cell-Death Ratio (%)
	LSEC		HSC	
Control	4 ± 1%	2 ± 0%	5 ± 1%	2 ± 1%
5-FU	6 ± 0%	3 ± 0%	5 ± 1%	5 ± 1%
IBU	8 ± 1%	2 ± 1%	3 ± 1%	5 ± 1%
RIF	5 ± 1%	3 ± 1%	5 ± 0%	3 ± 0%
	Kupffer		Hepatocytes	
Control	4 ± 0%	3 ± 1%	88 ± 1%	3 ± 1%
5-FU	2 ± 0%	12 ± 0%	87 ± 1%	4 ± 0%
IBU	2 ± 0%	12 ± 2%	88 ± 1%	2 ± 1%
RIF	3 ± 0%	8 ± 0%	88 ± 0%	2 ± 0%

Rifampicin (RIF), ibuprofen (IBU), 5-fluorouracil (5-FU), liver sinusoidal endothelial cells (LSECs) and human stellate cells (HSCs).

## Data Availability

The authors agree to make the data and materials supporting the results or analyses presented in their paper available upon reasonable request.

## Supplementary Materials

The following supporting information can be downloaded at https://www.mdpi.com/article/10.3390/pharmaceutics16050607/s1, Table S1: Hep-medium formulation; Table S2: Summary of liver markers in the two cell culture media used for liver model culturing.
