# Supplementary material for: In Vitro Human Liver Model for Toxicity Assessment with Clinical and Preclinical Instrumentation
Source: Pharmaceutics. 2024 Apr 29;16(5):607. doi: 10.3390/pharmaceutics16050607 (PMC11124512; doi:10.3390/pharmaceutics16050607)
Supplement: Supplementary file 1 [file pharmaceutics-16-00607-s001.zip › Supplementary Table S1.pdf]

Supplement Table S1. Hep medium formulation

Volume	Substance
**40 mL**	Williams E colourless
**5 mL**	Daily Vits™ Tablets (NOWFoods, USA) in 200 mL Milli-Q water and filtered through a 0.22 µm filter (Millipore, USA)
**5 mL**	One Total Amino tablet (G&G Vitamins, UK) solubilised in 5 mL of PBS (Merck KGaA, Germany)
**15 µL**	Human Insulin (Merck KGaA, Germany) (final concentration: 0.51 µmol/L)
**2 µL**	Fatty acids (FA) from oil digested with lipase (Merck KGaA, Germany), inactivated[17] and filtered through a 0.22 µm filter (Millipore, USA)
	The osmolality of the mixture was 387 mOSM, measured with Osmomat 030 (Gonotec, Germany). After two days of culturing the cells with this medium, the supernatant had an osmolality between 290-310 mOSM
