# Supplementary material for: In Vitro Human Liver Model for Toxicity Assessment with Clinical and Preclinical Instrumentation
Source: Pharmaceutics. 2024 Apr 29;16(5):607. doi: 10.3390/pharmaceutics16050607 (PMC11124512; doi:10.3390/pharmaceutics16050607)
Supplement: Supplementary file 1 [file pharmaceutics-16-00607-s001.zip › Supplementary Table S2.pdf]

Supplementary Table S2: Summary of liver markers in the two cell culture media used for liver model culturing.

**Clinical marker**	**Hep medium**	**Williams E medium**
Triglycerides (mmol/L)	0.02	0.1
Glucose (mmol/L)	9.4	10.67
Albumin (g/L)	0.5	3.4
ALP (U/L)	0	0
AST (U/L)	2	5
ALT (U/L)	2.6	1.5
GGT (U/L)	3.5	5.6

Alanine aminotransferase (ALT), aspartate aminotransferase (AST), gamma-glutamyl transferase (GGT), alkaline phosphatase (ALP), glucose, triglycerides, and albumin
